# Sarcopenia of the deltoid as an indicator of outcome following reverse total shoulder arthroplasty: a systematic review and meta-analysis

**DOI:** 10.1016/j.jseint.2025.05.035

**Published:** 2025-06-16

**Authors:** Liam O'Dwyer, Martin S. Davey, J. Tristan Cassidy

**Affiliations:** a School of Medicine, University of Galway, Galway, Ireland; b Department of Trauma & Orthopaedics, UHL Hospitals Group, Limerick, Munster, Ireland; c Department of Surgery, Royal College of Surgeons, Dublin, Ireland; d School of Medicine, University of Limerick, Limerick, Ireland

**Keywords:** Shoulder, Reverse, Arthroplasty, Sarcopenia, Radiology, Deltoid, Orthopedics

## Abstract

**Background:**

Reverse total shoulder arthroplasty (rTSA) is increasingly utilized for managing rotator cuff tear arthropathy, shoulder osteoarthritis, and proximal humerus fractures. The deltoid generates the key mechanical force after rTSA. The functional success of a rTSA is dependent on the status of the deltoid muscle. Sarcopenia, defined as age-related loss of skeletal muscle mass and function, has been linked to poor outcomes in various surgical contexts. This systematic review investigates the impact of radiologically measured deltoid sarcopenia on functional and patient-reported outcomes following rTSA.

**Methods:**

A systematic search of MEDLINE, PUBMED, and SCOPUS was conducted to identify studies assessing deltoid sarcopenia via radiological imaging in rTSA patients. Inclusion criteria included studies reporting both radiological measurements of the deltoid and functional or patient-reported outcome measures. Risk of bias was assessed using the Newcastle-Ottawa Scale and the Methodological Index for Nonrandomized Studies.

**Results:**

Fourteen studies comprising 1,681 shoulders were included. Studies ranged from 2010 to 2024. Eight studies used a prosthesis based on the Grammont concept design, four studies used a reduced neck shaft angle of 135°, and two studies in which the prosthesis was not stated. Radiological modalities included magnetic resonance imaging, Computed Tomography, ultrasound, and X-ray, with deltoid cross-sectional area being the most frequently measured parameter. Meta-analysis revealed a weak, nonsignificant association between preoperative deltoid cross-sectional area and postoperative Constant-Murley Score (r = 0.12, *P* = .702) but a moderate positive association with postoperative muscle strength (r = 0.38, *P* < .001). Deltoid sarcopenia, particularly fatty infiltration and reduced muscle caliber, was associated with poorer outcomes, including lower Constant-Murley Score, reduced strength, and diminished patient-reported outcome measures.

**Conclusion:**

Radiological evidence of deltoid sarcopenia correlates with suboptimal functional and patient-reported outcomes after rTSA, the pooled effects of which are not statistically significant. However, methodological heterogeneity and inconsistent use of sarcopenia metrics limit the generalizability of these findings. Standardized imaging protocols and sarcopenia definitions are essential for improving predictive accuracy and guiding clinical decision-making.

The number of reverse total shoulder arthroplasty (rTSA) procedures has grown exponentially in recent years, with it commonly being used in the treatment of conditions such as rotator cuff tear (RCT) arthropathy, shoulder osteoarthritis and proximal humerus fractures (PHFs).^2^ The concept of rTSA is based on transfer of the shoulder's center of rotation inferiorly and medially, allowing the deltoid muscle to have a greater mechanical advantage with a larger lever-arm in abduction. Therefore, it is understandable that global deltoid deficiency is a contraindication to rTSA, while partial deficiency is a relative contraindication.^3^ There is a paucity of literature examining the preoperative and postoperative status of the deltoid and outcome post-rTSA.

Sarcopenia has been defined as the age-related loss of skeletal muscle mass and function.^15^ Previous literature has shown that sarcopenia of the psoas muscle has been linked to an increased risk of complications and mortality in trauma patients.^16^ Although sarcopenia can be estimated clinically using grip strength or gait speed tests, gold standard sarcopenia assessment of muscle mass and quality tends to be estimated radiologically using magnetic resonance imaging (MRI), Computed Tomography (CT), Dual Energy X-ray Absorptiometry, or ultrasound (US).^15^

The aim of this systematic review was to identify radiological measurements of sarcopenia of the deltoid in patients who have undergone rTSA. Secondly, to identify metrics used to assess function and patient-reported outcome measures (PROMs) post-rTSA. A meta-analysis was performed to analyze the association between radiological measurements of sarcopenia (of the deltoid) and outcome (PROMs, function, and complications) post-rTSA. It was hypothesized that reduced size and quality of the deltoid (higher rates of sarcopenia) will correlate with significantly poorer outcomes in patients who undergo rTSA.

## Methods

### Search strategy

A systematic review was performed according to Preferred Reporting Items for Systematic Reviews and Meta-analyses (PRISMA) guidelines with a view to evaluate all articles that measure sarcopenia radiologically, in patients who have had rTSA. A comprehensive systematic search of MEDLINE, PUBMED, and SCOPUS was performed in November 2024. The population intervention comparison outcome (PICO) search strategy used can be seen in Table I. The following terms were used: (Reverse Shoulder Replacement OR Reverse Shoulder Arthroplasty OR rTSA OR reverse shoulder replacement) AND (Imaging OR Magnetic Resonance Imaging OR MRI OR Computed Tomography OR CT OR Dual Energy X-ray Absorptiometry OR DEXA OR DXA OR Ultrasound OR US) AND (Deltoid Sarcopenia OR Muscle Mass OR Muscle Density OR Cross Sectional Area OR CSA OR Volume OR Deltoid) AND (Outcomes OR Function OR Patient Reported Outcome Measures OR PROM). After duplicates were removed two independent reviewers (LOD and MSD) reviewed the list of articles for inclusion/exclusion criteria. Where there was disagreement between the two reviewers the articles were discussed with the senior author (JTC) for resolution.

**Table I:** Population intervention comparison outcome (PICO) search strategy.

PICO elements	Keywords	Search strategies
P (Population)	Patients undergoing rTSA	Reverse Shoulder Replacement OR Reverse Shoulder Arthroplasty OR rTSA OR RSR
I (Intervention)	Radiological imaging (eg, CT, MRI, DEXA, US) Sarcopenia	Imaging OR Magnetic Resonance Imaging OR MRI OR Computed Tomography OR CT OR Dual Energy X-ray Absorptiometry OR DEXA OR DXA OR Ultrasound OR US
C (Comparison)	Sarcopenia vs. Absence of sarcopenia	Deltoid Sarcopenia OR Muscle Mass OR Muscle Density OR Cross Sectional Area OR CSA OR Volume OR Deltoid
O (Outcome)	Postoperative outcomes	Outcomes OR Function OR Patient Reported Outcome Measures OR PROM

*rTSA*, reverse total shoulder arthroplasty; *CSA*, cross-sectional area; *DEXA*, Dual Energy X-ray Absorptiometry; *US*, ultrasound; *MRI*, magnetic resonance imaging; *PROM*, patient-reported outocme measures; *RSR*, reverse shoulder replacement.

### Inclusion criteria

Articles were included on the following basis: (1) patients who had rTSA, (2) patients who had preoperative or postoperative radiological imaging, (3) measurements of sarcopenia of the deltoid, and (4) studies which measured functional and/or PROMs.

### Exclusion criteria

Articles were excluded on the following basis: (1) shoulder hemiarthroplasty, (2) anatomic shoulder arthroplasty, (3) articles not in English language, (4) case reports, (5) no full text, and (6) studies with no imaging.

### Risk of bias

The Newcastle-Ottawa scale (NOS)^17^ was used to assess for the risk of bias. Studies were evaluated in the areas of selection (four items), comparability (1 item), and exposure (three items). Areas were scored out of 1 star for each item apart from comparability were two stars were available. Studies were scored out of nine. Previously used threshold's for NOS scoring were applied^22^: 7-9, good quality; 4-6, fair quality; and ≤3, poor quality. The NOS scored were independently assessed by author's LOD and MSD, a consensus was reached by consulting the senior author (JTC) for any disagreements.

### Assessment of quality of evidence

As this study included the evaluation of nonrandomized controlled trials, the methodological index for nonrandomized studies (MINORS)^14^ was used to evaluate quality of studies found. The MINORS scoring system is designed to evaluate not comparative and noncomparative studies in terms of methodological rigor. Comparative studies were graded out of 24 from 12 items, while noncomparative studies were graded out of 16 from 8 items. Articles were scored zero to two for each item (zero, not reported; 1, reported but inadequately; 2, reported adequately). Percentage-based thresholds were applied to MINORS scoring as follows: ≥12/16 or 18/24 (≥75%), good quality; 9-11/16 or 12-17/24 (50-75%), moderate quality; and ≤8/16 or 11/24 (≤50%), poor quality. The level of evidence (LOE) for each study was also determined according to the journal of bone and joint surgery.^19^ The study type was determined (Prognostic, diagnostic, therapeutic, or economic) followed by grading of LOE from I (highest) to IV (lowest). Both MINORS scoring and LOE were independently scored by author LOD and MSD, a consensus was reached by consulting the lead author (JTC) for any disagreements.

### Outcomes of interest and data extraction

General categories for outcomes of interest were predetermined with some specific outcomes. The following outcomes were recorded: (1) patients numbers and demographics (age and sex); (2) length of follow-up; (3) study characteristics and LOE; (4) patient cohort (cuff tear arthropathy [CTA], gleno-humeral osteoarthritis [GHOA], PHF, irreparable RCT, infection, and revision); (5) imaging modality; (6) timing of imaging (preoperative vs. postoperative); (7) axial segment from which images were analyzed; (8) outcomes measured within individual studies; and (9) prosthesis design/concept. Preoperative and postoperative imaging was included as the aim was to assess muscle status (deltoid size and/or quality) and determine its correlation with outcome/function. Heterogeneity of studies was taken into account for pooled analysis. Only studies utilizing the same variables for correlation were used to calculate pooled effects.

### Statistical analysis

Statistical analysis was performed using *JASP* (version 0.19.0; University of Amsterdam, Amsterdam, Netherlands). to determine relationships between variables. Significance level was set at *P* ≤ .05. Fischer's z-transformation and variance were calculated in excel. Transformed data including Fischer's z-score and variance were used to calculate pooled effect sizes in JASP. Within JASP the meta-analysis module was used with restricted maximum likelihood used to estimate parameters in a random effects model.

## Results

### Literature search

**Figure 1:**
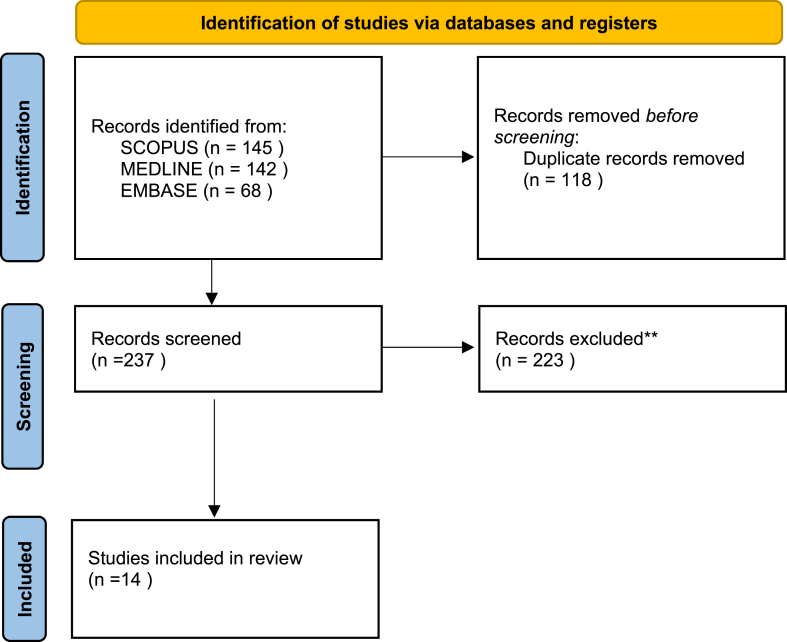
A PRISMA flow diagram for the search strategy used, including identification, screening, and studies included. *PRISMA*, Preferred Reporting Items for Systematic Reviews and Meta-analyses.

The literary search according to PRISMA guidelines returned 355 articles in total. There were 145 articles found on SCOPUS, 142 articles on PUBMED, and 68 from MEDLINE. Upon removal of 118 duplicates there were 237 articles for review. Following application of inclusion and exclusion criteria 14 studies were included in this systematic review. The PRISMA flow diagram for the search can be seen in Figure 1.

### Patient demographics

There were 1,695 shoulders from 14 studies, with an average of 121 ± 201 shoulders per study. The largest study had 799 patients while the smallest study 23 shoulders. The mean age of the patients was 71.53 ± 2.87 (37-90) with 1 study not reporting patient age. There was a mean follow-up of 32.85 ± 20.85 (5.75-158) months, with two studies not reporting mean follow-up time. There were 292 males from 806 patients in the 12 studies that reported sex, with an average of 37% ± 9.96% (21%-54.55%) being male. Two studies did not report sex. There were 9 studies that gave an exact breakdown of the indication for rTSA. The other studies 799 were ‘primary rTSA’, 211 elective (no breakdown), 30 rotator-cuff (RC) intact and 77 RC deficient, 59 CTA or GHOA, 30 CTA or RCT. The studies which provided information on indication comprised 489 shoulders cross those 9 studies. The largest cohort were CTA (n = 271), followed by PHF (n = 113), GHOA (n = 55), RTC (n = 39), and failed hemiarthroplasty (n = 11). Eight studies utilized the Grammont style medialized prosthesis with 155° neck shaft angle, four studies utilized a lateralized prothesis design with 135° neck shaft angle, two studies did not state the prosthesis design used, more information can be seen in Table II. Studies included ranged from 2010 to 2024.

**Table II:** A summary of the fourteen studies included in this systematic review highlighting patient numbers (n), imaging modality, timing of imaging (preoperative vs. postoperative), cohort (trauma vs. elective), quantitative measurements as indicators of sarcopenia, quantitative measurements as indicators of sarcopenia, the axial segment from which measures were taken from CT and MRI, and outcomes that were measured in each study.

	Patients (n)	Prosthesis/concept	Imaging modality	Preop vs. postop	Cohort	Quantitative	Qualitative	Axial segment	Outcomes measured
De Boer et al^1^ (2022)	59	Aequalis (Grammont concept)	MRI or CT	Both (35 pre; 58 post)	CTA or GHOA	Deltoid volume (mathematical)	-		CMS; FE; ER; abduction; strength; OSS
Fischer (2017) et al^5^	64	Grammont concept	US	Postop	31 CTA, 18 GHOA, 9 failed hemi, 6 PHF	Deltoid caliber	-		Above/below average
Fischer (2020) et al^4^	35	Grammont concept	US	Preop	32 CTA, 3 PHF	Deltoid caliber	-		ASES; FE; abduction
Greiner et al^6^ (2010)	23	Delta III reversed prosthesis DePuy/Grammont	MRI (18) or CT (5)	Postop	17 CTA, 4 PHF, 2 failed hemi	Relative contrast	Goutallier	Infraglenoid rim	CMS
Hill et al^7^ (2023)	25	N = 23: Glenoid: Zimmer TM extended posthumerus: Zimmer TM humeral stem N = 1: Glenoid: Tornier/Stryker Reverse II extended central posthumerus: Tornier/Stryker Ascend Flex humeral stem N = 1: Glenoid: Tornier/Stryker Reverse II extended central posthumerus: Zimmer TM humeral stem	CT	Both	10 GHOA, 15 CTA	CSA	-	Center of humeral head; head/neck junction; metadiaphyseal junction	ASES; FE; ER
Hung et al^8^ (2021)	88	Zimmer Trabecular Metal Reverse Shoulder System. Inlay implant NSA of 155	MRI	Preop	61 CTA, 27 GHOA	-	Goutallier	Midglenoid	ASES
Jeon and Rhee^9^ (2018)	59	Aequalis (NSA 155)/grammont concept	MRI	Preop	59 CTA	CSA	-	Coracoid tip	FE
Mcclatchy et al^10^ (2022)	107	Medial genoid, lateral humerus, Biomet Comprehensive shoulder system (NSA 135/145)	MRI (64) or CT (43)	Preop	30 RC intact, 77 RC deficient	Deltoid volume (sum of 5 slices)	-	24 mm from most inferior aspect of acromion	ASES; FE; ER; SANE
Nakazawa et al^11^ (2023)	60	Aequalis ascend flex (NSA 135)	MRI	Both	31 CTA, 29 RCT	CSA	Goutallier	Tip of GT. Midpoint of deltoid	CMS; strength
Panzica et al^12^ (2017)	100	Not available	CT	Preop	100 PHF	CSA	Goutallier	Superior rim of the glenoid. Coracoid process. Inferior rim of the glenoid	-
Rajabzadeh-Oghaz et al^13^ (2024)	799	Equinoxe (Exactech) NSA 135	MRI	Preop	799 primary rTSA	Flatness; 3D volume/Scapula; Atrophy; Sphericity; Hounsfeld	-		CMS; ASES; Pain VAS; FE; ER; IR; abduction; global shoulder function; max weight in hand
Wiater et al^18^ (2015)	30	Zimmer trabecular metal reverse shoulder system Inlay implant NSA of 155	CT	Preop	30 CTA or RTC	CSA; fatty infiltration	Goutallier	Largest Humeral Head	CMS; ASES; SST/SSV; FE; ER; IR; ADLs; strength
Wu et al^20^ (2024)	211	Not available	X-ray	Preop	Elective (no breakdown)	Deltoid size; DHR; Subcutaneous tissue size; HS	-		LOS; operative time; PJI
Yoon et al^21^ (2017)	35	Aequalis, NSA 155	MRI	Preop	25 CTA, 10 RTC	3D volume/BMI	-		CMS; ASES; SST/SSV; pain VAS; FE; ER; IR

*rTSA*, reverse total shoulder arthroplasty; *NSA*, neck shaft angle; *MRI*, magnetic resonance imaging; *CT*, Computed Tomography; *CSA*, cross-sectional area; *CMS*, Constant-Murley Score; *RC*, rotator-cuff; *RCT*, rotator cuff tear; *US*, ultrasound; *CTA*, cuff tear arthropathy; *GHOA*, gleno-humeral osteoarthritis; *PHF*, proximal humerus fracture; *3D*, three-dimensional; *ASES*, American Shoulder and Elbow Surgeons; *BMI*, body mass index; *FE*, forward elevation; *ER*, external rotation; *HS*, distance from humeral head to skin; *postop*, postoperative; *preop*, preoperative; *IR*, internal rotation; *SANE*, Single Assessment Numeric Evaluation; *OSS*, Oxford Shoulder score; *PJI*, prosthetic joint infection; *VAS*, visual analogue scale; *SST/SSV*, subject shoulder test/value; *DHR*, deltoid-radius-to-humeral head radius ratio; *NSA*, neck shaft angle; *ADL*, activities of daily living; *LOS*, length of stay.

### Imaging and outcomes

There were five studies which used MRI exclusively, three which used CT exclusively, three studies used either MRI or CT, two which used US, and 1 study which x-ray. There were 12 studies that used preoperative imaging, five used postoperative imaging, and among these three studies used both preoperative and postoperative. Quantitative measures of sarcopenia used include cross-sectional area (CSA; five studies), three-dimensional volume; deltoid size (x-ray); deltoid-humerus ratio (x-ray); deltoid caliber (US). One qualitative measure of sarcopenia was identified (Goutallier’s). Axial slices chosen for CSA measurement included the following: (1) superior tip of the glenoid; (2) infraglenoid rim; (3) midglenoid point; (4) coracoid process; (5) center of the humeral head; (6) 24 mm from the most inferior aspect of the acromion; (7) humeral head/neck junction; (8) humerus metaphyseal/diaphyseal junction; and (9) midpoint of the deltoid. There were 18 different outcomes measured. The outcome measured the most was shoulder forward elevation measured eight times, followed by American Shoulder and Elbow Surgeons (ASES) measured seven times, followed by Constant-Murley Score (CMS) and external rotation both measured 6 times. One study by Panzica et al^12^ did not measure any outcomes.

### Risk of bias, quality assessment, and level of evidence

Results can be seen in Table III. Risk of bias was assessed using the NOS scoring system. The mean NOS was 6/9 ± 1.29 (4/9-8/9). From the 14 studies included in this review, seven could be interpreted as ‘good quality’ and seven as ‘fair quality’. No study was interpreted as ‘poor quality’. Study quality was assessed using MINORS scoring. All 14 studies were noncomparative studies and were thus graded out of 16. The mean MINORS score was 11/16 ± 1.60 (9/16-13/16). There were five studies graded as ‘good quality’ and nine studies graded as ‘moderate quality’. No study was graded as ‘poor quality’. Among the studies, there were 10 prognostic studies, three therapeutic, and 1 diagnostic. There was three prospective studies and 10 retrospective studies. There were seven level III studies, six level IV studies, and 1 level II study. There were no studies at level I and V.

**Table III:** Summarizing risk of bias assessment using Newcastle-Ottawa scale (NOS) with its interpretation, quality assessment using methodological index for nonrandomized studies (MINORS) and interpretation and level of evidence (LOE), and study design.

	NOS score	Interpretation	MINORS score	Interpretation	LOE	Study design
De Boer et al^1^	7/9	Good	13/16	Good	III	Prognostic
Fischer (2017) et al^5^	7/9	Good	12/16	Moderate	IV	Diagnostic
Fischer (2020) et al^4^	8/9	Good	13/16	Good	II	Prognostic
Greiner et al^6^	6/9	Fair	11/16	Moderate	IV	Prognostic
Hill et al^7^	4/9	Fair	9/16	Moderate	IV	Prognostic
Hung et al^8^	5/9	Fair	9/16	Moderate	IV	Prognostic
Jeon and Rhee^9^	5/9	Fair	9/16	Moderate	III	Prognostic
Mcclatchy et al^10^	5/9	Fair	9/16	Moderate	III	Prognostic
Nakazawa et al^11^	6/9	Fair	11/16	Good	IV	Prognostic
Panzica et al^12^	5/9	Fair	9/16	Moderate	IV	Prognostic
Rajabzadeh-Oghaz et al^13^	8/9	Good	12/16	Good	III	Prognostic
Wiater et al^18^	6/9	Good	9/16	Moderate	III	Prognostic
Wu et al^20^	8/9	Good	12/16	Good	III	Prognostic
Yoon et al^21^	6/9	Good	10/16	Moderate	III	Prognostic

### Correlation between preoperative deltoid measures and outcomes

One study found deltoid CSA was found to be significantly weakly associated with postoperative muscle strength (r = 0.33)^11^ with no significant association found with CMS. Similarly, no significant association between deltoid volume and CMS was found.^1^ Another study found deltoid CSA to be positively associated with postoperative CMS (q = 0.432), ASES (q = 0.377), improvement in ASES (q = 0.493), and strength (q = 0.454).^18^ Deltoid volume as calculated by the average of CSA at three levels was found to be higher in patients with satisfactory outcomes vs. unsatisfactory, but this was not significant.^9^ Patients with excellent or good anterior shoulder elevation were categorized as ‘satisfactory outcome’. Total deltoid volume, calculated as a sum of five CSA segments, was significantly associated with satisfactory FE.^10^ When analyzing individual portions of the deltoid volume, the posterior deltoid was significantly associated with satisfactory ASES score. The normalized deltoid volume (three-dimensional deltoid volume/scapula volume) was significantly associated with greater abduction and lower ASES scores in females, with no association in males.^13^ Another study adjusted deltoid volume for body mass index (BMI), they found deltoid volume/BMI to be significantly positively associated with CMS (r = 0.525), FE (r = 0.402), and ER (r = 0.334).^21^ Quantitative measures of fatty infiltration using Hounsfield units found significant associations with postoperative FE and strength.^13^ Higher grades of fatty infiltration, as measured using Goutallier classification, were found to be significantly associated with lower ASES functional subscores.^8^ There was no association found with ASES composite score and pain subscore. However, in another study, postoperative ASES was found to be significantly associated with fatty infiltration (q = −0.401).^18^ Deltoid caliber, as measured by US, found a significant positive association with both FE (r = 0.445) and abduction (r = 0.373).^4^ One study using x-ray found larger deltoid size to be associated with higher infection risk (OR: 4.52, 95% confidence interval [CI]: 1.50-13.60), longer operative time (beta = 0.164), and longer LOS (beta = 0.332).^20^ However, distance from humeral head to skin was a stronger predictor of all three variables, with higher infection risk (OR 4.70), longer operative time (beta = 0.234), and longer length of stay (beta = 0.432). A summary of the these results can be found in Table IV.

**Table IV:** A summary of the studies which correlated preoperative deltoid size or quality with postoperative function.

	Measure associated	CMS	ASES	Strength	Pain VAS	Forward elevation	External rotation	Internal rotation	Abduction	Other
De Boer et al (2022)^1^	Deltoid volume vs.	β = 0.062 [−0.024 to 0.148]	-	-	-	-	-	-	-	-
Fischer et al (2020)^4^	Deltoid caliber					∗r = 0.445		-	∗r = 0.373	
Hung et al (2021)^8^	Deltoid Goutallier class		Composite ∗Functional subscore Pain subscore				-			
Jeon and Rhee (2018)^9^	Deltoid CSA					Satisfactory group vs. unsatisfactory				
Mcclatchy et al (2022)^10^	Deltoid volume		Cuff intact Cuff deficient			Cuff intact ∗Cuff deficient	Cuff intact Cuff deficient	-		SANE Cuff intact *P* = .987 Cuff deficient *P* = .153
Nakazawa et al (2023)^11^	Deltoid area	*P* = −.187		∗*P* = .331						
Rajabzadeh-Oghaz et al (2024)^13^	Normalized deltoid volume	No association	∗significant association among females	No association	∗significant association among males	No association	No association	No association	∗significant association among females	
	Deltoid fat percentage	∗significant association	∗significant association	∗significant association	No association	∗significant association	No association	∗significant association	No association	
Wiater et al (2015)^18^	Total deltoid volume	∗*P* = .432	∗*P* = .377	∗*P* = .439	*P* = −.318	*P* = .212	*P* = −.161	*P* = −.035	-	∗SSV: *P* = .427 ADLs: *P* = .218
Wu et al (2024)^19^	Deltoid size	-	-	-	-	-	-	-	-	∗LOS: *P* = .364 ∗Operative time: 0.172 ∗PJI: 0.260
Yoon et al (2017)^21^	Deltoid muscle volume	∗*P* = .525	*P* = .205		*P* = −.254	∗*P* = .402	∗*P* = .334	*P* = −.294	-	SST: *P* = .252

*CMS*, Constant-Murley Score; *ASES*, American Shoulder and Elbow Surgeons; *VAS*, visual analogue scale; *SANE*, Single Assessment Numeric Evaluation; *SSV*, subject shoulder value; *ADL*, activities of daily living; *LOS*, length of stay.

∗ Denotes significant relationship.

### Correlation between postoperative deltoid measures and outcomes

One study found deltoid volume, as measured by US, was significantly positively associated with CMS (b = 0.049) and abduction strength (b = 0.006).^1^ Two studies measured CSA. In one of these studies, percentage change in deltoid CSA compared with preoperative CSA, as measured using CT, found no correlation with ASES, FE, or ER.^7^ The other study looking at CSA, as measured by MRI, found a significant moderate positive association with muscle strength at 2 years postoperative (r = 049) and no significant association with CMS.^11^ Another study found fatty infiltration, as measured by Goutallier classification, was significantly negatively associated with CMS and age-related CMS.^6^ Deltoid muscle caliber, as measured by US, was significantly positively associated with above average outcomes (ASES ≥76) post-rTSA.^5^

### Meta-analysis

**Figure 2:**
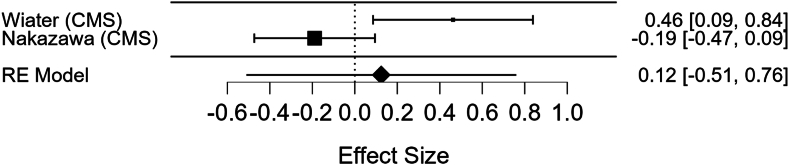
Forest plot showing the relationship between preoperative deltoid cross-sectional area (CSA) and postoperative Constant-Murley Score (CMS), highlighting individual study correlations and pooled effect sizes.

A meta-analysis of two studies (Wiater and Nakazawa) was conducted to assess the relationship between preoperative deltoid CSA and CMS and strength (Table V). For CMS, the pooled effect size was weak and not statistically significant (Fisher's z = 0.124, 95% CI: [−0.51, 0.12], *P* = .702). When back-transformed, the correlation coefficient (r) was approximately 0.12, indicating a very weak positive association. However, substantial heterogeneity was observed (Q = 7.334, *P* = .007; I^2^ = 86.365%), suggesting variability between studies. Individual study results were inconsistent, with Wiater reporting a moderate positive correlation (r = 0.43) and Nakazawa reporting a weak negative correlation (r = −0.19). These findings can be visualized in the forest plot labelled Figure 2.

**Table V:** Meta-analysis results summarizing the relationships between deltoid cross-sectional area (CSA) and postoperative outcomes, including Constant-Murley Score (CMS) and strength.

	CMS	Strength
Study one		
Nakazawa et al 2023^11^	r = −0.187 *P* = .193	∗r = 0.331 *P* = .019
Study two		
Wiater et al (2015)^18^	∗q = 0.432 *P* = .017	∗q = 0.454 *P* = .015
Fischer's pooled effects	z = 0.124, 95% CI: [−0.51, 0.12], *P* = (0.702).	z = 0.397 95% CI: [0.170, 0.624], *P* < .001
Heterogenicity	Q = 7.334, *P* = .007; I^2^ = 86.365%	Q = 0.367, *P* = .545; I^2^ = 0.000%
Correlation coefficient	r = 0.12	r = 0.38
Interpretation	Weak positive	Moderate positive

*r*, Pearsons correlation coefficient; *q*, Spearman's rank correlation coefficient; *z*, Fischer z-transformation; *CI*, confidence interval; *Q*, Cochran's Q; *I*^*2*^, heterogeneity.

### For postoperative muscle strength

**Figure 3:**
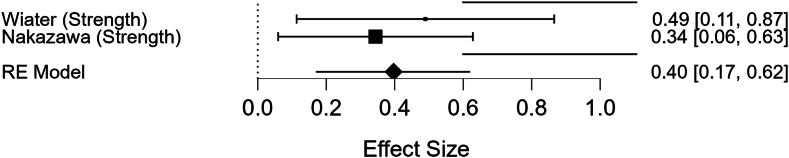
Forest plot showing the relationship between preoperative deltoid cross-sectional area (CSA) and postoperative muscle strength, highlighting individual study correlations and pooled effect sizes.

The pooled Fisher's z -transformed effect size was 0.397 (95% CI: [0.170, 0.624], *P* < .001), which corresponds to a moderate positive correlation (r = 0.38). No significant heterogeneity was detected (Q = 0.367, *P* = .545; I^2^ = 0.000%), suggesting consistent findings between the studies. These results indicate a moderate and reliable relationship between preoperative deltoid CSA and postoperative Strength. These findings can be visualized in the forest plot labeled Figure 3.

## Discussion

The most important finding in this study is that meta-analysis findings suggest that there is correlation, which was not statistically significant, with preoperative deltoid CSA and postoperative functional outcomes. Although this was not found to be statistically significant, the results appear to be underpowered and represents an area that warrants further study.

The findings of this systematic review suggest that radiological markers of deltoid sarcopenia negatively influence outcomes after rTSA, although the magnitude of the effect varies depending on the metric used and the study methodology. Deltoid CSA, the most commonly reported parameter, showed inconsistent correlations with functional outcomes such as the CMS and patient-reported measures. This variability underscores the need for a standardized approach to defining and measuring sarcopenia in rTSA patients.

MRI emerged as the preferred imaging modality due to its superior resolution for soft tissue evaluation, although CT remains widely utilized for preoperative planning. CT's utility is limited postoperatively by metal artifact interference, which compromises its accuracy. CT also subjects patients to radiation without providing any clinical benefit postoperatively. Preoperative imaging, integrated into routine clinical workflows, offers a practical opportunity to assess sarcopenia without additional patient burden.

The meta-analysis highlighted a moderate correlation between preoperative deltoid CSA and postoperative strength, emphasizing the functional relevance of muscle mass preservation. Similarly, qualitative markers such as fatty infiltration were consistently associated with poorer outcomes, supporting the hypothesis that muscle quality is as critical as quantity in determining surgical success.

However, significant heterogeneity in study designs, imaging techniques, and outcome measures complicates interpretation. For example, studies varied in their choice of axial slices for CSA measurements, with no consensus on the most clinically relevant region. Furthermore, the inconsistent reporting of demographic variables, such as BMI, may confound the observed associations, as sarcopenia's impact can vary by patient phenotype.

The contrast between populations undergoing rTSA further complicates the interpretation of these results. One population can be labelled as ‘elective’, who undergo rTSA for CTA/GHOA, can be subject to months of rehab to improve the quality of the deltoid. The PHF population does not have this luxury. The radiological condition of their deltoid may lead to decisions around surgery vs. conservative management, or the rehabilitation programs prescribed to them. The majority of studies included in this review do not comment on the status of the rotator cuff. However, McClatchy et al^10^ found an association between function and deltoid size in patient who were cuff deficient but not in those with intact cuffs. Cuff status is another variable that can influencing the importance of the deltoid postoperatively.

Clinically, these findings highlight the importance of preoperative identification of sarcopenia to optimize patient selection and potentially modify surgical techniques. While rTSA remains viable for patients with partial deltoid insufficiency, those with severe sarcopenia may benefit from targeted prehabilitation strategies or alternative interventions.

Future research should aim to standardize imaging protocols and define thresholds for sarcopenia that are predictive of outcomes in rTSA. Longitudinal studies examining the trajectory of muscle changes postoperatively and their relationship to clinical recovery will further refine our understanding of sarcopenia's role in rTSA success.

### Limitations

This systematic review and meta-analysis has several limitations that warrant consideration. The included studies demonstrated significant variability in methodologies, including imaging modalities, measurement techniques for sarcopenia, and outcome assessment tools. This heterogeneity limited the ability to directly compare results across studies and may have introduced bias. In addition, cuff status and preoperative function were not always stated in the included papers; this is an additional variable that can affect postoperative function and must be kept in mind when interpreting these results. There is neither consensus on the best radiological metrics, for assessing deltoid sarcopenia, nor on standardized axial slices or imaging protocols. This inconsistency complicates the interpretation and generalization of results. Most studies were nonrandomized and retrospective, with no Level I evidence available. The quality of evidence, as assessed by NOS and MINORS scores, ranged from fair to good, but the lack of robust prospective data diminishes the strength of conclusions. Individual studies often had small sample sizes, which may have limited statistical power and introduced variability in reported outcomes.

The studies included a variety of indications for rTSA, as well as varying proportions of males and females, which may influence the generalizability of findings to specific subgroups undergoing rTSA. Preoperative imaging was the primary focus, with limited studies assessing changes in deltoid morphology postoperatively. This restricts the ability to understand the dynamic impact of sarcopenia on recovery and long-term outcomes. Many studies did not control for confounding factors such as BMI, comorbidities, or physical activity levels, which are known to influence muscle quality and surgical outcomes. The use of diverse functional and PROMs across studies, such as the CMS and ASES score, may result in inconsistencies and make pooling results challenging. There is a potential for publication bias, as studies with significant findings may be more likely to be published, potentially overestimating the association between deltoid sarcopenia and postoperative outcomes.

## Conclusion

Radiological evidence of deltoid sarcopenia correlates with suboptimal functional and patient-reported outcomes after rTSA, the pooled effects of which are nonstatistically significant. However, methodological heterogeneity and inconsistent use of sarcopenia metrics limit the generalizability of these findings. Standardized imaging protocols and sarcopenia definitions are essential for improving predictive accuracy and guiding clinical decision-making.

## Disclaimers:

Funding: No funding was disclosed by the authors.

Conflicts of interest: The authors, their immediate families, and any research foundation with which they are affiliated have not received any financial payments or other benefits from any commercial entity related to the subject of this article.
